# Exploring Cellular Heterogeneity: Single-Cell and Spatial Transcriptomics of Alzheimer Disease Brains and iPSC-Derived Microglia

**DOI:** 10.21203/rs.3.rs-5045715/v1

**Published:** 2024-10-16

**Authors:** Anjali Garg, Sheeny Vo, Logan Brase, Ekaterina Aladyeva, Ricardo D’O. Albanus, Aasritha Nallapu, Hongjun Fu, Oscar Harari

**Affiliations:** Department of Psychiatry, Washington University, Saint Louis, St. Louis, Missouri, United States of America; Department of Neuroscience, OSU Wexner Medical Center, The Ohio State University, Columbus, OH, United States of America; Department of Psychiatry, Washington University, Saint Louis, St. Louis, Missouri, United States of America; Department of Psychiatry, Washington University, Saint Louis, St. Louis, Missouri, United States of America; Department of Psychiatry, Washington University, Saint Louis, St. Louis, Missouri, United States of America; Department of Psychiatry, Washington University, Saint Louis, St. Louis, Missouri, United States of America; Department of Neuroscience, OSU Wexner Medical Center, The Ohio State University, Columbus, OH, United States of America; Department of Psychiatry, Washington University, Saint Louis, St. Louis, Missouri, United States of America

**Keywords:** Alzheimer Disease, spatially resolved transcriptomics, single-nucleus RNA-seq, integration, amyloid beta, Tau, microglia, immunostaining

## Abstract

**Background:**

Substantial evidence has established the critical role of microglia, the brain’s resident immune cells, in the pathogenesis of Alzheimer’s disease (AD). Microglia exhibit diverse transcriptional states in response to neuroinflammatory stimuli, and understanding these states is crucial for elucidating the underlying mechanisms of AD.

**Methods:**

In this work, we integrated single-cell and spatially resolved transcriptomics data from multiple cohorts and brain regions, including microglia from experimental and human brains.

**Results:**

This comprehensive atlas revealed a great heterogeneity of microglial states, with a significant enrichment of specific states, including activated microglia, in AD brains compared to controls. Further integration of spatial transcriptomics and immunohistochemistry showed that activated microglia are predominantly located in the external cortical layers near amyloid plaques, while homeostatic microglia are more prevalent in the internal cortical layers and further away from the plaques. These spatial patterns were further validated using P2RY12 immunostaining, which confirmed the reliability of the transcriptomic data.

**Conclusion:**

By integrating single-cell and spatial transcriptomics, we have provided a detailed atlas of microglial diversity, revealing the regional and pathological specificity of microglial states.

## Background

Alzhreimer’s disease (AD) is a complex and devastating neurodegenerative condition affecting millions globally. It is characterized by the accumulation of amyloid beta (Aβ) plaques, neurofibrillary tangles, gliosis, and neuronal death, leading to cognitive impairment and dementia(1).

Microglia plays a significant multidimensional role in AD, contributing to inflammation, phagocytosis, and neurodegeneration(2). Microglia, the brain’s resident immune cells, exhibit diverse morphology, physiology and gene expression patterns, which is associated with specific phenotype (3–5). Experiments shows induced pluripotent stem cell-derived microglia-like cells (iPSC-derived MLC) have significant potential in modeling microglial behavior in vitro, offering new avenues for studying AD pathogenesis (6–8).

Moreover, high-throughput sequencing technologies, particularly single-cell RNA-sequencing (scRNA-seq) and spatially resolved transcriptomics (SRT) enable high-resolution mapping of cellular states and spatial distributions within tissues, providing unprecedented insights into AD pathogenesis. Identifying and characterizing microglia transcriptional states holds great promise for understanding the underlying mechanisms of this neurodegenerative disease(9, 10).

While the pathological hallmarks of AD, such as Aβ plaques and misfolded tau proteins, are well-documented, several fundamental questions remain unanswered. These include which microglial transcriptional states are associated with early and late-onset Alzheimer’s, how these states are distributed in the human brain, and how they relate to Aβ plaques and tau tangles. Additionally, understanding the predominant transcriptional states across different cortical layers that correlate with high Aβ plaque and tangle burden could provide critical insights into disease progression. Similarly, it is critical to understand how experimental models *in vitro* recapitulate transcriptional states observed in *ex vivo* tissues. To address these questions, we study microglial transcriptional states across human and experimental model samples in AD.

We integrated microglial single-cell datasets from human and experimental models to capture novel insights of the heterogeneity of pathways, biological processes, and microglia transcriptional states present in AD pathology. This approach allowed identification of different transcriptional states associated with various stages of AD, such as Sporadic AD (sAD), presymptomatic, early, and late-AD carriers, also autosomal dominant AD (ADAD), shedding light on the diversity of immune responses and molecular signatures in the context of the AD. Moreover, the integration of cortical layers from human middle temporal gyrus (MTG) AD samples with single-cell atlas data has revealed the distribution of specific microglial states in different cortical layers, providing a spatial understanding of the immune response in relation to the pathological features of the disease. In addition, we validated this specific pattern by measuring the distribution of homeostatic microglial marker (i.e. P2RY12) and Aβ plaque via the co-immunofluorescence staining in human AD brains.

In summary, our findings emphasize the power of leveraging diverse single-cell and SRT datasets to advance our understanding of the molecular mechanisms underlying AD. These insights have implications for the development of targeted therapeutic approaches aimed at modulating neuroinflammatory processes and potentially altering the course of the disease. By serving as an atlas to translate findings from experimental models to postmortem human brain studies and vice versa, this research bridges the gap between basic science and clinical application, paving the way for developing new therapeutic strategies in AD.

## Methods

### Integration the single cell transcriptomics profile across studies

The first dataset was collected from the transcriptome of human wild-type TREM2 and isogenic TREM2-R47H DAM xenografted microglia (xMGs), isolated from chimeric AD mice(11). The second dataset, integrated with xenografted mic was collected from multimodal CRISPR-based genetic screens in human iPSC-derived microglia(12). The third single-nuclei transcriptomic profiles of brains were collected from the Knight-ADRC and DIAN brain banks (WASHU) to enrich with the carriers of familial mutations causal of early onset familial AD (in *APP, PSEN1* and *PSEN2*), carriers of high-risk variants in TREM2, and sporadic non-carriers AD and controls. SnRNA-seq data pre-processing, clustering, and cell state annotation were described elsewhere(13). The last dataset integrated into these three datasets was derived from the aged postmortem brain samples from the Religious Orders Study and Rush Memory and Aging Project (ROSMAP) study(14). Samples with minimal number of cells 10 were considered for further analysis.

### Integration of cohorts

The selected four scRNA-seq data underwent a rigorous preprocessing pipeline, adhering to best practices in data normalization and integration. The merged expression matrix was normalized using the *SCTransform* protocol by Seurat Version 5.0.1(15). This function calculates a model of technical noise in scRNA-seq data using “regularized negative binomial regression” as described previously in ref(16). We regressed out, during the normalization, the number of genes, the number of UMIs, and the percentage of mitochondria.

After extensive optimization of Seurat V5 integration methods we found Anchor-based CCA integration was outperformed. The principal components were calculated using the first 3000 variable genes, and the Uniform Manifold Approximation and Projection (UMAP) analysis was performed with the top 30 PCs. However, PC 1 with high batch effects was excluded from downstream analysis. The clustering was done using a resolution of 0.3.

### Sample and batch entropy by clusters

To evaluate the sample and batch effects in clustering, Shannon entropy was used to calculate individual cluster entropies (Eq. 1)(17), and the weighted sum was used to calculate overall clustering entropy (Eq. 2). The entropies were normalized by dividing each entropy value by the maximum entropy possible for each scenario (batch (*n* = 4) = −2; sample (n = 490 = −8.9).


1 $$H(i)=-\sum_{j\in K}\;p\left(i_{j}\right){\;log}_{2p}\left(i_{j}\right)$$

2 $$H=-\sum_{i\in C}\;H(i)\frac{N_{i}}{N}$$

### Pointwise mutual information

To quantify the association between the original transcriptional state labels with integrated clusters, Pointwise mutual information (PMI) was calculated for each cohort (Eq. 3). We normalized the PMI value between − 1 to 1, where value of −1 indicates a complete absence of association between the original transcriptional state labels and integrated clusters, 0 denotes independence, and + 1 signifies a perfect alignment where each label consistently corresponds to a specific cluster, and vice versa. We used the svs version 3.0.0 to calculate PMI.


3 $$npmi\;(x;\;y)=\frac{pmi(x;\;y)}{h(x,\;y)}$$

### Microglia transcriptional state differential expression

To determine if there was unique functionality of each integrated cluster, we fitted a linear mixed model that predicted the expression level of each gene for the individual nuclei by integrated cluster and corrected for the subject of origin and datasets (Eq. 4)(18). Expression levels were extracted from the Seurat objects using *GetAssayData* with the ‘slot’ parameter set to “counts”. The R package nebula (v1.5.1)(19) was used to implement the model, including parameters for a zero-inflated negative binomial distribution (model = “NBLMM”, method = “LN”) and the random effect of the subject of origin(19). The number of UMI’s per nuclei was already accounted for during *SCTransformation*, so the model did not need to account for the number of UMI’s. Within each microglia cluster, differential expression (DE) was calculated on each cluster versus all other.


(4) Expression~Cluster+(1|Subject);ZImodel=~1;ZINBdistribution

### Pathway analyses

The upregulated genes (logFC > 0.25) identified for each integrated cluster were utilized in a subsequent pathway analysis employing the R-based application enrichR. For pathway enrichment, we utilized gene sets from either “KEGG 2021 Human” or “GO Biological Process 2021”.

A heatmap was constructed to consolidate the upregulated “GO Biological Process 2021” (GO_BP) hits across all clusters. The −log10 P values for each GO_BP term within each cluster were combined into a single table. The top ten GO_BP terms with the highest averages of transformed P values across all clusters were then ranked. Subsequently, these terms were processed through rrvgo (v 1.14.1), an R package implementing the Revigo(20) tool for summarizing GO_BP terms. The calculateSimMatrix function was employed to determine the relationships between the GO_BP terms, with parameters set as follows: orgdb = “org.Hs.eg.db”, ont = “BP”, method = “Rel”. The terms were further summarized using reduceSimMatrix with the following specifications: score = “Rank”, threshold = 0.6, orgdb = “org.Hs.eg.db”. The summarized terms or “parentTerms” along with their corresponding P-values were then utilized to generate the heatmap. Row ordering was performed using the dist function with method = “euclidean”, while column ordering utilized method = “p” for Pearson correlation.

### Differential proportion analysis

We employed linear regression models testing each individual’s cluster compositions to identify associations between microglia transcriptional states and disease group. More explicitly, the number of nuclei a subject had in a specific cluster was divided by the subject’s total nuclei count for that cluster creating a proportion. The proportions were normalized using a cube root transformation and were corrected by sex and disease status depending on the variable of interest. We removed participants without sample information. We utilized *glm*, a standard function in R, to implement the model (Eq. 5).


(5) (Proportion)1/3~Sex+ADstatus

To visualize these results, cell state proportions were averaged between the samples in a group and displayed in a stacked barplot using the ggplot2 (v3.4.4) library in R.

### Integration of snRNA-Seq and SRT data from human MTG

The 10x Genomics Visium data from the MTG of three Control (Braak stages I-II, male, aged 58, 72, and 82 years) and three AD (Braak stages III-IV, male, aged 86, 86, and 89 years) cases was collected(21). We specifically chose the gray matter from three AD cases for integration due to its direct relevance to AD pathology. The merged expression matrix of these three AD samples underwent normalization using the SCTransform protocol in Seurat Version 5.0.1, with parameters set to assay = “Spatial” and method = “poisson”. Principal component analysis was conducted on the first 3000 variable genes, followed by UMAP analysis utilizing the top 12 principal components. Clustering was performed using default parameters (0.8). We employed ‘Reference-based’ integration method of Seurat V5 to integrate snRNA-seq and SRT dataset. Subsequently, using above mentioned four datasets we constructed an integrated reference comprising ~ 222K single-cell profiles of microglial transcriptional states. This integrated reference facilitated the analysis of conserved states across different conditions and brain regions. Given the pivotal role of Homeostatic and Activated transcriptional states in AD etiology, we deliberately selected these states for further in-depth investigation.

### Annotation of cortical layers and AD pathology-associated spots

To compare Homeostatic and Activated transcriptional states expression between cortical layers, we calculated the fraction by dividing the number of spots in a specific transcriptional state by the total number of spots in that cortical layer. This data was utilized to create a heatmap using the ggplot2 (v3.4.4) library in R.

Aβ plaques and AT8-tangles spots were categorized into proximal and distal levels based on their distance from Aβ and tau pathology respectively. All spots were previously manually annotated regarding the presence of Aβ plaques based on immunostaining from immediately adjacent tissue slices(21). For the comparisons between Aβ-proximal versus distal spots, we defined as Aβ-proximal spots any spot that directly overlapped Aβ plaques, and others as Aβ-distal spots.

We identified and quantified Homeostatic and Activated transcriptional state expression changes around these regions by selecting spots with the top 25% highest probability for each level. We conducted a Chi-square significance test in R to compare the fraction between levels. Specifically, we divided the number of spots in a specific transcriptional state by the total number of spots in that level to create a fraction. The results were visualized using bar plots created with the ggplot2 (v3.4.4) library in R.

### Immunostaining

Human brain frozen sections were air dried 60°C for 10 min and then fixed and permeabilized by prechilled acetone at − 20°C for 15 min followed by antigen retrieval(21). After the sections were blocked for 1 hours by 10% donkey serum in 1xPBS, the sections were incubated overnight at 4°C with primary antibody P2RY12 (1:1000). On the following day, the sections were washed three times with 1x PBS and then incubated with secondary antibodies for 2 hours at 37°C, followed by nuclei staining with Hoechst33342 and autofluorescence quenching. The slides were mounted using Fluoromount-G Mounting Medium and imaged with a Zeiss Axio Observer microscope. We used a statistical t-test to compare the P2RY12 staining between the AD samples.

## Results

### Transcriptional diversity across human and iPSC-derived microglia atlas

To provide a compressive repertoire of microglia diversity in postmortem human brains and human cell based experimental models we integrated single-cell and SRT dataset from multiple cohorts and brain regions. Table 1 provides a summary of the clinical, neuropathological and experimental data of these datasets.

**Figure 1:**
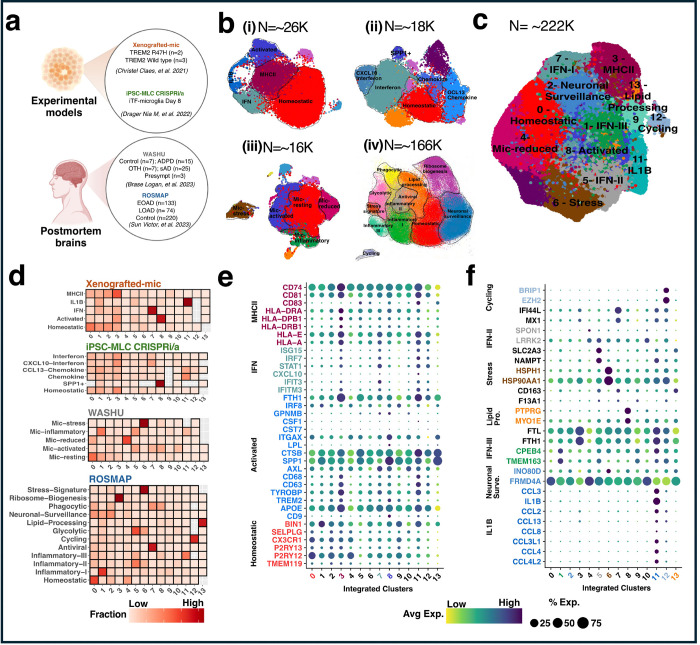
Integration of Single-cell transcriptomics datasets. **a** Diagram depicting the single-cell transcriptomics dataset utilized. **b** Highlighted transcriptional states selected from each single-cell transcriptomics dataset, demarcated with dotted lines. **c** UMAP plot showing 14 distinct integrated clusters labeled 0–13, comprising a total of 222,822 cells. **d** Quantification of individual cell state contributions to the integrated transcriptional state. e-f) Gene expression analysis within each transcriptional state, referencing studies by Yun Chen et al. (2021) and Sun et al. (2023). Cluster numbers and gene names are highlighted with the same color code to indicate enrichment.

We harmonized microglia from two *in vitro* models: The first experimental dataset involves Xenografted-microglia (xMGs) that represents seven distinct clusters of approximately ~26K single cell nuclei(11). In this experiment, wild-type and R47H human microglial progenitors were transplanted into 5x-hCSF1 mice, to investigate how this mutation affects lipid droplet accumulation, and the microglial response to Aβ plaques. After seven months, GFP-expressing TREM2 and isogenic TREM2-R47H xMGs were extracted from the brains of 5x-hCSF1 mice and subjected to scRNA-seq(11). Each cluster represents a unique microglial state for both TREM2 and TREM2-R47H xMGs(11) (Fig 1a, Table 1a). We also included in this atlas multimodal CRISPR-based genetic screens in human iPSC-derived microglia(12) (Fig 1a). Unsupervised clustering and UMAP dimensional reduction of iTF-Microglia revealed nine transcriptionally distinct clusters for ~18K nuclei(12). Our atlas also integrated *ex vivo* data. We included microglia from postmortem human microglia from parietal cortex from our previous work(13). We identified nine microglia transcriptional states for about 16K nuclei from 57 donors, including 15 carriers of pathogenic mutations in *APP* and *PSEN1* (autosomal dominant AD; ADAD), 25 sAD non-carriers. In addition, we included three individuals who matched AD-neuropathological criteria but without clinical cognitive impairment at death (presymptomatic), 7 individuals who matched non-AD neurodegenerative pathologies criteria (Other), and 7 individuals who exhibited neither neurodegenerative pathology nor evidence of dementia (Control) (Fig 1a, Table 1b). The last dataset includes ~166K single microglia nuclei from aged postmortem brain samples of 427 individuals from the ROSMAP study(14). This includes 207 AD donors (133 early and 74 late) and 220 control individuals across six brain regions: prefrontal cortex (PFC), hippocampus, mid-temporal cortex, angular gyrus, entorhinal cortex, and thalamus. Here, we identified 12 distinct microglia transcriptional states (Fig 1a, Table 1c).

We selected clusters that were sufficiently represented in each of the datasets (Fig 1b), considering both the number of cells contained within each cluster and the extent to which these clusters fairly represented the diverse range of donors included in the study. This selection process ensured that each cluster provided a comprehensive overview of the biological diversity present across the samples. By maintaining broad biological representation, we were able to maximize the significance and robustness of the integration, thereby enhancing the reliability and validity of our findings (**Additional file 1**). After comparing methods and optimizing parameters, we chose the Canonical Correlation Analysis (CCA) method implemented in the SeuratV5 package that high accuracy and exhibited minimal batch effects (**Fig S1a-h**). We harmonized a total of 222,822 nuclei into 14 clusters that are represented in the four datasets (Fig 1c). We confirmed that all samples and batches were adequately represented across the different clusters (**Fig S2a-b**).

We examined the contribution of each cohort’s transcriptional states in these integrated clusters. Cells from all cohorts were reorganized while preserving their biological relevance. (Fig 1d, **see NMI in Methods: Fig S1**). For instance, the majority of homeostatic cells from both *in vitro* and *ex vivo* were grouped into Cluster 0, stress-related cells from both *ex vivo* were found in Cluster 6 while activated states from *in vitro* and the *ex vivo* parietal cortex cohort converged into Cluster 8. Although no activated cluster was clearly identified in the Human multiple region (ROSMAP) samples(14), by virtue of aggregating multiple dataset we were able to reassign few nuclei to the activated cluster (Fig 1d). Finally, to evaluate the association of transcriptional states to amyloid plaques and tau tangles, we selected SRT datasets from human Middle temporal gyrus (MTG) AD samples (N=3) generated using the 10X genomics Visium platform.

### Characterization of integrated transcriptional states

We annotated the 14 integrated clusters based on their molecular signatures and functions, utilizing well-known canonical standard markers for Homeostatic, Activated, IFN, and MHCII (22)(**Additional file 2**). We expanded this list by incorporating additional representative markers from Sun et al. (2023)(14) for newly reported transcriptional states. We identified an average of 941 upregulated genes (FDR <0.05) for each transcriptional, revealing a rich transcriptional diversity within all integrated transcriptional states (**Additional file 3**).

Homeostatic microglia (Cluster 0) were characterized by high expression of well-known homeostatic markers such as P2RY12 (log2FC = 0.28, adj. *p* = 7.73 × 10^−504^), P2RY13 (log2FC = 0.21, adj. *p* = 5.24 × 10^−131^), and CX3CR1 (log2FC = 0.19, adj. *p* = 9.23 × 10^−165^) (Fig 1e, **Additional file 3**). Activated microglia (Cluster 8) exhibited higher expression of SPP1 (log2FC = 1.45, adj. *p* = 7.97 × 10^−3290^), CD9 (log2FC = 1.83, adj. *p* = 2.27 × 10^−1331^), and TREM2 (log2FC = 0.59, adj. *p* = 1.50 × 10^−511^) (Fig 1e, **Additional file 3**).

In MHCII (Cluster 3), we observed higher expression of HLA-A (log2FC = 0.22, adj. *p* = *1.52* × 10^−182^), HLA-E (log2FC = 0.08, adj. *p* = *4.57* × 10^−42^), and HLA-DPB1 (log2FC = 0.64, adj. *p* = *6.17* × 10^−529^) and marker genes associated with “positive regulation of lymphocyte proliferation” (adj. *p* = *1.75* × 10^−2^) (Fig 1e, **Additional file 3 and 4**). In MHCII (Cluster 3), we observed higher expression of HLA-A (log2FC = 0.22, adj. *p* = *1.52* × 10^−182^), HLA-E (log2FC = 0.08, adj. *p* = *4.57* × 10^−42^), and HLA-DPB1 (log2FC = 0.64, adj. *p* = *6.17* × 10^−529^) and marker genes associated with “positive regulation of lymphocyte proliferation” (adj. *p* = 1.75 × 10^−2^) (Fig 1e, **Additional file 3 and 4**).

We identified three inflammatory states showing high expression of IFN-induced gene (Clusters1, 5, and 7). Cluster 7 showed overexpression of IFIT3 (log2FC = 1.57, adj. *p* = 1.37× 10^−1418^) (Fig 1e, **Additional file 3**), and showed strong functional enrichment in immune responses, including defense response to virus (adj. p = 7.45 × 10^−20^), and cytokine-mediated signaling pathway (adj. *p*= 2.77× 10^−11^) (**Additional file 4**). We also found TMEM163 (log2FC = 0.52, adj. *p* = 9.82 × 10^−483^) in Cluster 1 and LRRK2 (log2FC = 0.36, adj. *p* = 1.45 × 10^−125^) in Cluster 5 (Fig 1f, **Additional file 3**).

We observed significant enrichment of stress markers such as HSP90AA1 (log2FC = 1.73, adj. p = 5.92 × 10^−6228^) and HSPH1 (log2FC = 1.56, adj. p = 3.21 × 10^−3248^) related to the regulation of cellular response to heat (adj. p = 3.23 × 10^−8^) in Cluster 6 (Fig 1f, **Supplementary Tables 3 and 4**). Interleukin IL1B (log2FC = 3.70, adj. p = 8.41 × 10^−2568^) and CCL4 (log2FC = 4.82, adj. p = 1.31 × 10^−1480^) and additional genes related to the “cytokine-mediated signaling” (adj. p = 2.29 × × 10^−10^) were overexpressed in Cluster 11 (Fig 1f, **Supplementary Tables 3 and 4**).

Cluster 4 showed expression of SPARC (log2FC = 0.51, adj. *p* = 1.27× 10^−55^) (Fig 1e, **Additional file 3**), and strong functional enrichment of extracellular structure organization (adj. *p* = 2.68 ×10^−6^), external encapsulating structure organization (adj. *p* = 2.68 ×10^−6^), extracellular matrix organization (adj. *p* = 5.64 ×10^−4^), and ECM-receptor interaction (adj. *p* = 8.17×10^−4^), henceforth designated as Extracellular Vesicular (EV) microglia (**Additional file 4**). And Cluster 2 exhibited high expression of neurotransmitter receptors related to cell-cell junction organization (adj. *p* = 2.73 × 10^−3^) (Fig 1f, **Additional file 4**), while we identified Cluster 12 as a cycling state due to the enrichment in DNA metabolic process (adj. *p* = 2.42 × 10^−31^) (Fig 1f, **Additional file 4**). We annotated Cluster 13 as a lipid-processing state based on the enrichment of representative marker TPRG1 (log2FC = 1.20, adj. *p* = 9.96 × 10^−19^) (Fig 1f, **Additional file 3**). We did not find any specific functional enrichment in clusters 9 and 10, neither were those nuclei flagged by our quality control process. Therefore, we labeled them as Unknown (Fig 1e-f, **Additional file 3**).

**Figure 2:**
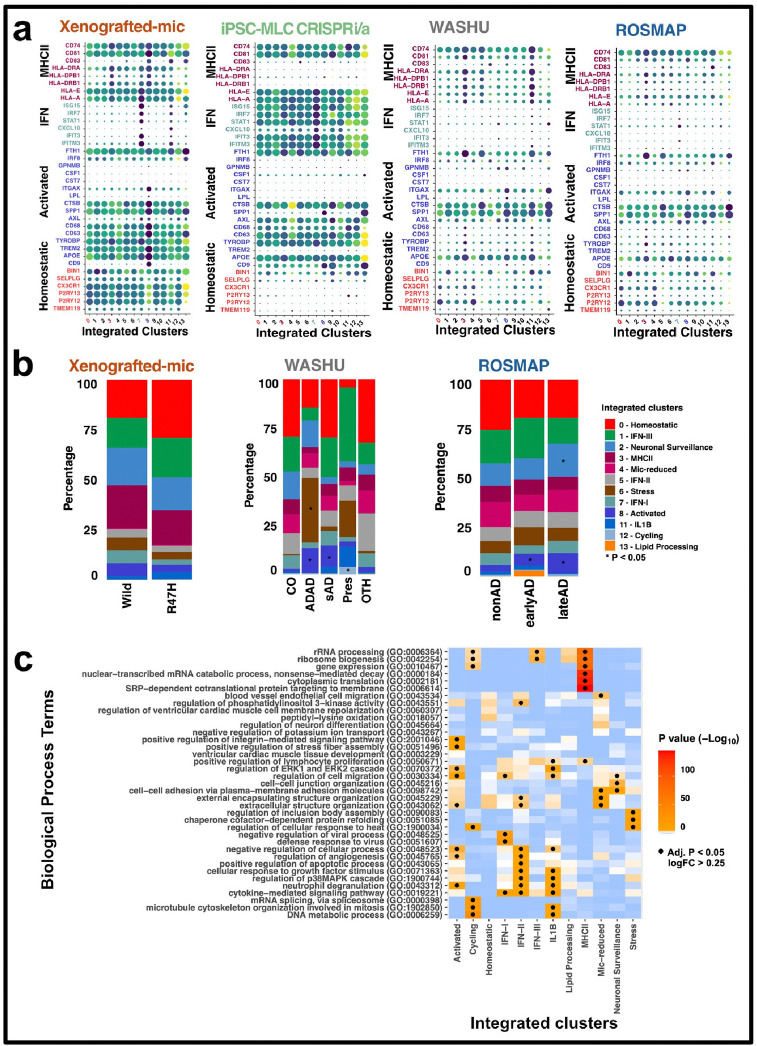
Biological conservation of functionality across diverse populations or conditions. **a** Analysis of conical marker gene expression within each integrated cohort. Cluster numbers and gene names are highlighted with the same color code to indicate enrichment. **b** Proportion plots illustrating the enrichment of specific transcriptional states in Control participants compared to all other participants across each cohort. Proportions were calculated for each sample (see Methods). For visualization, sample proportions are averaged by sample status. (*) denotes significant (p < 0.05) enrichment of that cluster within samples, as determined by linear regression. Exact p-values can be found in the Supplementary Dataset. ADAD: Autosomal Dominant Alzheimer’s Disease, sAD: Sporadic Alzheimer’s Disease, Pres: Presymptomatic, CO: Neuropathology-free, OTH: Non-Alzheimer’s Disease Neurodegenerative. **c** Heatmap displaying enriched pathways within the upregulated genes for each transcriptional state. Differentially Expressed Genes (DEGs) were identified from linear mixed models comparing each transcriptional state to all other states. Gene Ontology (GO) Biological Process terms were summarized and selected as described in the Methods. (·) indicates a significant (Benjamini–Hochberg adjusted p < 0.05) association, as calculated by the R package enrichR. Exact p-values can be found in the Source Data file.

Furthermore, we examined the expression of each marker gene in each dataset (Fig 2a and **Fig S3**), to verify they were consistently expressed. Our analysis revealed consistency in gene expression patterns across all datasets, further enhancing the reliability and generalizability of our insights. In summary, Clusters 0, 3, 8, 6, and 11 were classified as Homeostatic, MHCII, Activated, Stress, and IL1B. Based on strong evidence of gene expression and proportional analysis, Clusters 0, 3, 8, 6, and 11 were classified as Homeostatic, MHCII, Activated, Stress, and IL1B. IFN-I, IFN-II, and IFN-III cells were identified in clusters 7, 5, and 1. Clusters 2, 4, 12, and 13 were annotated as Neuronal Surveillance, extracellular vesicular, Cycling, and Lipid Processing respectively (Fig 1 c). The biological pathway analysis of each annotated transcriptional state is shown in **Additional file 5**.

### Relative proportions of microglia transcriptional states across AD groups

To evaluate whether the relative proportions of these transcriptional states change in AD, we tested the statistical significance of cell fractional differences between control and other individuals, including those in ex vivo datasets and in Xenografted-mic with AD risk variants in *TREM2* and wildtype (Fig 2b, **Additional file 6**).

A prominent Stress cell state was enriched in ADAD samples from the Human parietal cortex (WASHU) cohort (β = 0.30, *p* = 1.67 × 10^−2^; Fig 2b, **Additional file 6**). The Gene Ontology analysis revealed its association with “regulation of cellular response to heat” (adj. *p* = 3.29 × 10^−8^), “chaperone cofactor-dependent protein refolding” (adj. *p* = 1.15 × 10^−7^) and “regulation of inclusion body assembly” (adj. *p* = 6.19× 10^−4^) (Fig 2c, **Additional file 7**). These nuclei overexpressed HSP90AA1 and HSPH1 genes, suggesting these play a role in synaptic homeostasis and Aβ pathology, as previously observed in the entorhinal cortex in AD(23). In the case of Human multiple regions (ROSMAP) we did not find significant enrichment of Stress transcriptional state in AD groups comparison to control.

Both *ex vivo* datasets exhibited enrichment of Activated cell states in AD groups, but such association was not significant in the Xenografted TREM2-R47H samples. The Activated transcriptional state was specific to ADAD (β = 0.23, p = 1.78 × 10^−3^; Fig 2b, **Additional file 6**), and sporadic AD (β = 0.19, *p* = 5.53 × 10^−3^; Fig 2b, **Additional file 6**) in the Human parietal cortex (WASHU), similarly in the earlyAD (β = 0.12, *p* = 9.44 × 10^−12^; Fig 2b, **Additional file 6**) and late AD (β = 0.19, *p* = 9.81 × 10^−17^; Fig 2b, **Additional file 6**) from the Human multiple region (ROSMAP) cohort. Several experimental models support that in response to Aβ pathology, microglia cells proliferate, and acquire an activated transcriptional profile(24–26). However, excessive microglia activation may exacerbate neuronal damage at later stages of amyloid pathology(24,27–29). A gene ontology analysis reveals that activated cells are associated with the regulation of cell migration (*p* = 1.67 × 10^−4^), regulation of ERK1 and ERK2 cascade (*p* = 1.18 × 10^−2^), and positive regulation of stress fiber assembly (*p* = 2.61 × 10^−3^) (Fig 2c, **Additional file 7**). Additional studies support that the ERK1/2 pathway governs inflammatory cytokine production and iNOS expression in activated microglia, highlighting its importance in neuroinflammatory processes and as a potential therapeutic target (30). Additionally, activation of the Rho/ROCK signaling pathway boosts stress fiber assembly in microglia, facilitating essential cytoskeletal rearrangements for phagocytosis and activation (31,32).

The Cycling transcriptional state was enriched in Presymptomatic individuals (general linear regression β = 0.19, *p* = 1.86 × 10^−2^; Fig 2b, **Additional file 6**) from the Human parietal cortex (WASHU). Biological pathway analysis revealed associations of the Cycling transcriptional state with the DNA metabolic process (*p* = 2.42 × 10^−31^), microtubule cytoskeleton organization involved in mitosis (*p* = 1.12 × 10^−19^), and mRNA splicing via spliceosome (*p* = 1.32 × 10^−16^) (Fig 2c, **Additional file 7**). These findings suggest a potential role for these pathways in driving microglial proliferation, implicating their involvement in the early stages of neuroinflammatory processes associated with AD.

Neuronal surveillance cells were enriched in late onset AD (general linear regression β = 0.06, *p* = 2.06 × 10^−2^; Fig 2b, **Additional file 6**) samples from multiple region cohorts, showing it significant involvement in “cell-cell adhesion via plasma-membrane adhesion molecules” (*p* = 9.00 × 10^−4^), “cell-cell junction organization” (GO:0045216) (*p* = 2.73 × 10^−3^), and “regulation of cell migration” (*p* = 4.52 × 10^−2^) (Fig 2c, **Additional file 7**). These processes suggest a potential role in modulating immune cell infiltration, microglial activation and possibly involvement in the blood-brain barrier integrity, highlighting them as potential therapeutic targets for mitigating neuroinflammation in AD(33–36).

### Microglial transcriptional shift in response to AD pathology

**Figure 3:**
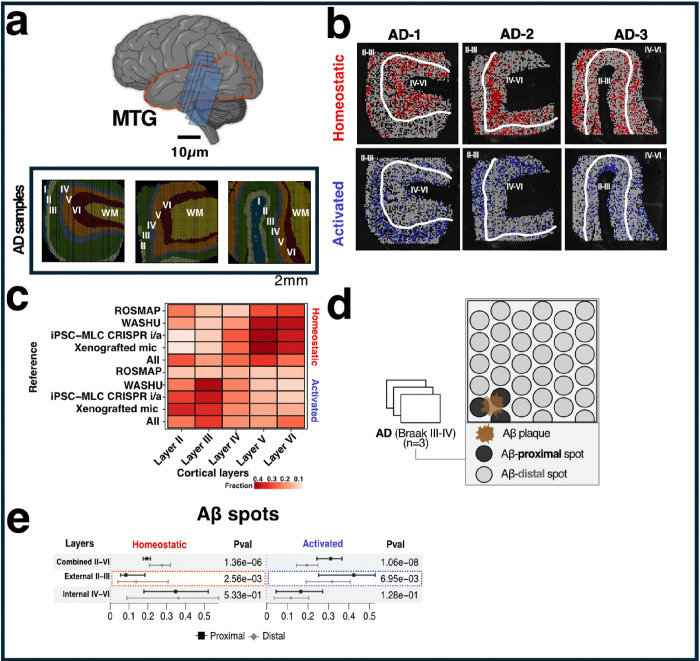
Microglial transcriptional shift in response to AD pathology. **a** Spatial transcriptomics (SRT) of the middle temporal gyrus (MTG) in Alzheimer’s disease (AD), with each section being 10 μm thick. **b** Visium spots highlighting the top 25% highest probability in red for Homeostatic states and in blue for Activated states. **c** Heatmap illustrating the fraction of predicted transcriptional states within each cortical layer. **d**Overview of spatial transcriptomics Aβ localization. Aβ-proximal spots refer to those directly overlapping Aβ plaques, while all others are considered Aβ-distal. **e** Upper: Quantification of transcriptional states around proximal and distal Aβ spots for Combined II-VI, External II-III, and Internal IV-VI cortical layers. Chi-square significance tests were used to calculate p-values.

We integrated SRT datasets from human MTG AD samples (N=3) generated using 10X genomics Visium platform(21). We selected those spots characterizing the cortical layers II to VI obtaining a total of 7,884 spots. As few spots were captured for layer I, we removed those from our analyses. Thus, we integrated this data with the scRNA-seq datasets(11–14) to investigate the distribution of microglia transcriptional states among cortical layers (Fig 3a) and their proximity to AD pathological lesions (Aβ or neurofibrillary tangles (NFT)). To achieve this, we conducted a reference-based integration to deconvolve and assign scores to each spot based on the resemblance of microglial transcriptional states. In our analysis, we focused on the highly predicted (top 25%) Visium spots for each AD pathology, examining the distribution of Homeostatic and Activated transcriptional states across cortical layers (Fig 3b), and dataset (**Fig S4**).

We observed a significant increase in spots capturing Activated microglia in the external cortical layers II-III (p=2.20e-16), while internal layers IV-VI showed an increase in Homeostatic signatures (p<2.2e-16) (Fig. 3c, **Additional file 8).** We discarded that this was not driven by Aβ or NFT. However, in the case of the Human multiple regions (ROSMAP) reference, we were unable to capture significant Activated spots (p=5.24e-01).

Furthermore, we analyzed the relationship of Activated and Homeostatic transcriptional states in relation to the proximity of amyloid plaques (identified using Rabbit anti-Aβ antibody) and NFT (identified using Human/murine phospho-tau pSer202/Thr205 antibody) (Fig. 3d). We identified that spots proximal to plaques (<55 microns) tended to show Activated microglia (*p*=1.06e-08), while distal spots showed a predominance of Homeostatic microglial expression (*p*=1.36e-06). The presence of the microglial Activation state in proximity to amyloid plaques was predominantly observed in external cortical layers II and III (*p*=2.56e-03), while the Homeostatic state was predominantly observed distal to amyloid plaques in external cortical layers II and III (*p*=6.95e-03) (Fig. 3e, **Additional file** 9). Notably, no such distinction was observed in relation to NFT (**Fig S5**). To ensure that our observations were not driven by a single AD sample and/or limited to integrated data, we examined the distribution of transcriptional states across all AD samples using each cohort as a reference (**Fig S6, Additional file 9**). We found a prevalence of microglial Activation states near amyloid plaques, and the Homeostatic state was predominantly observed distal to amyloid plaques in each sample. Importantly, this distribution pattern was not driven by differences in the burden of amyloid plaques among cortical layers.

### Validation of homeostatic microglia distributions in the cortical layers of human MTG

We performed immunochemistry experiments to validate the finding from the transcriptional studies. We stained for P2RY12, a unique marker for homeostatic microglia to map and analyze the distribution and activity of microglia in the adjacent 10 μm section of human MTG, which is 10 μm interval from the Visium gene expression section(21). This technique leverages antibodies that specifically bind to the P2RY12 protein, a purinergic receptor predominantly expressed in homeostatic microglia. We performed P2RY12 and Aβ co-immunostaining to assess the spatial relationship of homeostatic microglia with Aβ plaques, although the immunostaining of P2RY12 and Aβ plaque was not on the same section with Visium gene expression assay.

**Figure 4:**
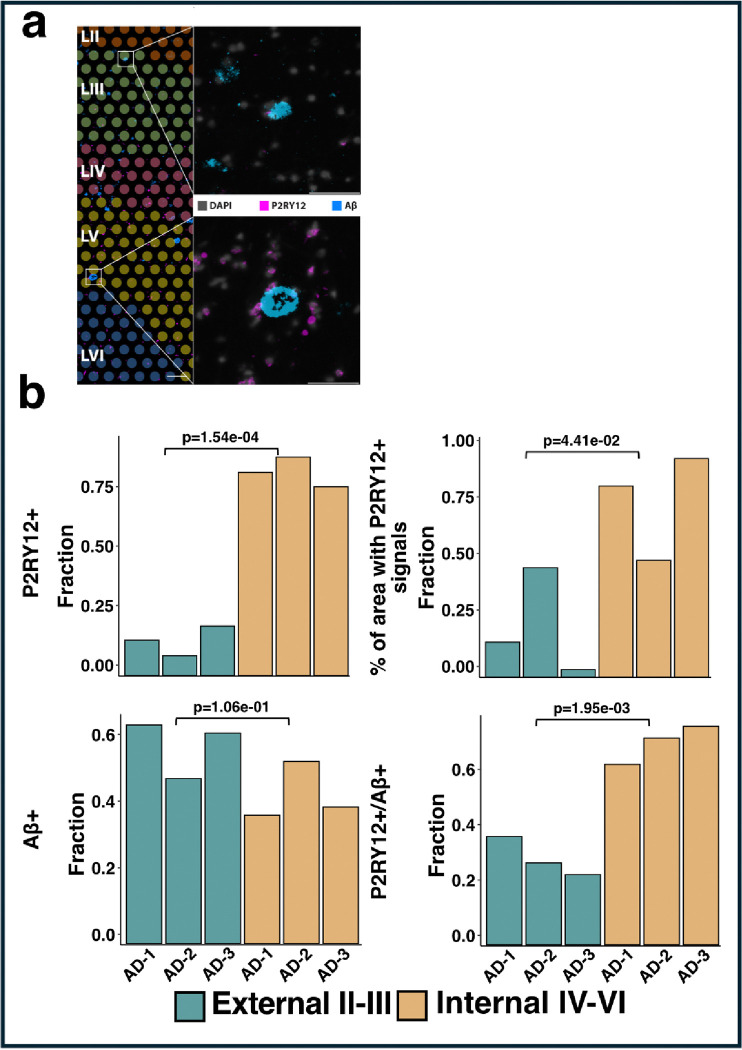
Immunostaining of P2RY12 gene across AD samples. **a** Immunofluorescence (IF) staining of P2RY12 and Aβ on adjacent sections (10 μm interval) from the middle temporal gyrus of a human AD sample was aligned with 10x Genomics Visium spots (color coded) at layers II-VI (Chen et al. ANC, 2022). High-mag images of IF staining of nuclei (DAPI, grey), homeostatic microglia (P2RY12, magenta) and AD pathological hallmark (Aβ plaques, blue) in external layers II-III (top panel) and internal layers IV-VI (bottom panel) were selected. Scale bar, 200 μm for layers II-VI image, 50 μm for external and internal high-mag images. **b** Bar plots comparing the counts of IF stained P2RY12+ cells and Aβ+ spots distribution in cortical layers II-III and IV-VI across all AD samples. Upper Left: P2RY12+ count, Upper Right: Aβ+ spots, Lower Left: P2RY12+/Aβ+ overlapped, Lower Right: P2RY12+/Aβ− spots. To compare the P2RY12+ count or/and Aβ+/− spots between layers II-III and IV-VI for all AD samples, counts in each group were normalized with the total number in layers II-VI. p-values were calculated using a two-sided t-test. Exact p-values can be found in the Source Data file.

Our registration of the immunostaining image with H&E staining on the Visium gene expression sections shows that the distribution of P2RY12-positive (+) counts (p = 1.54e-04) and the percentage of the area with P2RY12+ signals (p = 4.41e-02) across the internal cortical layers IV-VI, as observed through single-cell and SRT data (Fig. 4b, **Additional file 10**). The distribution of Aβ+ spots (p = 1.06e-01), as well as the overlap of P2RY12+ with Aβ+ spots (p = 1.95e-03) among cortical layers, further confirmed that this distribution was not driven by amyloid plaques (Fig. 4b, **Additional file 10**).

## Discussion

The integration of single-cell and SRT data from various cohorts and brain regions has provided a comprehensive repertoire of microglia diversity in postmortem human brains and human cell-based experimental models. This study highlights the importance of considering the multifaceted nature of microglial dynamics in AD(37). We harnessed data from different *in vitro* models, including xenografted-microglia (xMGs) and human iPSC-derived microglia, and integrating it with *ex vivo* human microglia from the ROSMAP study and parietal cortex samples, to integrate them into a microglial transcriptional atlas. This approach has enabled the identification of distinct microglial states associated with Homeostatic, Activated, IFN-mediated, and MHCII-expressing states across datasets.

However, the literature is limited in exploring how these varying transcriptional states correlate with Aβ plaques and NFT, and how this relationship may differ across regional or cortical areas. Our findings reveal significant alterations in microglial transcriptional states in regions associated with AD pathology, particularly exacerbated in cortical layers II and III. This regional specificity underscores the nuanced interplay between microglial states and AD pathology and cerebral cortex anatomy. Activated microglial states were predominantly localized to the outer cortical layers, whereas Homeostatic states were more prevalent in the inner cortical layers. This distribution pattern was consistent across all AD samples, indicating a robust association between microglial states and cortical layer-specific pathology.

Furthermore, our study uncovered intriguing associations between microglial states and Aβ plaque proximity. To the best of our understanding, this is the first study to show that regions proximal to Aβ plaques exhibited higher Activated microglia in human brains, whereas distal regions displayed a prevalence of Homeostatic states. This spatial relationship suggests a dynamic microglial response to Aβ pathology, where microglia near plaques are more likely to be in an Activated state, potentially phagocyting Aβ but also contributing to local neuroinflammatory processes.

The elucidation of diverse transcriptional states of microglia and their spatial distribution in relation to AD pathology provides valuable insights into the complexity of microglial responses in neurodegenerative diseases. These findings suggest potential therapeutic avenues for modulating microglial function in AD. For instance, strategies aimed at balancing microglial activation across cortical regions may help mitigate neuroinflammation and neuronal damage associated with AD. Additionally, targeting specific transcriptional states of microglia could offer more precise therapeutic interventions.

However, it is necessary to consider certain limitations of this study. The reliance on a limited number of spatial transcriptomics (N = 3 AD samples) may restrict the generalizability of our findings. Similarly, our analysis was confined to the middle temporal gyrus (MTG), which may not capture the full spectrum of microglial diversity across different brain regions. Additionally, the inability to capture significant Activated spots in the Human multiple-region (ROSMAP) reference dataset highlights the need for further validation through larger sample sizes and more diverse reference datasets. Future studies should aim to validate these findings thoroughly and explore the therapeutic potential of modulating specific microglial states in AD.

## Conclusion

This work significantly contributes to our understanding of microglial activity in Alzheimer’s disease. By integrating single-cell and SRT datasets, we have provided a detailed atlas of microglial diversity, revealing the regional and pathological specificity of microglial states. The study underscores the importance of considering the multifaceted nature of microglial dynamics in neurodegenerative diseases and highlights potential therapeutic targets for modulating microglial function in AD. Future research should focus on validating these findings in larger and more diverse cohorts to enhance their translational potential.

## Data Availability

The single nucleus data from the Knight ADRC accessed in this study are found in the National Institute on Aging Genetics of Alzheimer’s Disease Data Storage Site (NIAGADS) with accession number NG00108 [https://www.niagads.org/datasets/ng00108]. The raw single nucleus data from the DIAN brain bank accessed in this study are available under restricted access to maintain individual and family confidentiality. These samples contain rare disease-causing variants that could be used to identify the participating individuals and families. Access can be obtained by request through the online resource request system on the DIAN Website:https://dian.wustl.edu/our-research/for-investigators/dian-observational-study-investigator-resources/. The ROSMAP single nucleus RNA sequencing data used in this study are available at Synapse under Synapse ID syn52293417 [https://www.synapse.org/#!Synapse:syn52293417]. Source data is provided with this paper. The xenografted microglia (xMGs) single cell RNA sequencing data used in this study was access from XX as described in xxx. CRISPR-based genetic screens in human iPSC-derived microglia used in this study available on NCBI GEO, accession number GSE178317.

## References

[1] Esin RG, Safina DR, Khakimova AR, Esin OR. Neuroinflammation and Neuropathology. Neurosci Behav Physiol. 2022;52(2):196–201. 35317271 10.1007/s11055-022-01223-5 PMC8930459

[2] Miao J, Ma H, Yang Y, Liao Y, Lin C, Zheng J, Microglia in Alzheimer’s disease: pathogenesis, mechanisms, and therapeutic potentials. Front Aging Neurosci. 2023 Jun 15;15:1201982. 37396657 10.3389/fnagi.2023.1201982 PMC10309009

[3] Wolf SA, Boddeke HWGM, Kettenmann H. Microglia in Physiology and Disease. Annu Rev Physiol. 2017 Feb 10;79(1):619–43. 27959620 10.1146/annurev-physiol-022516-034406

[4] Holtman IR, Skola D, Glass CK. Transcriptional control of microglia phenotypes in health and disease. Journal of Clinical Investigation. 2017 Jul 31;127(9):3220–9. 28758903 10.1172/JCI90604 PMC5669536

[5] Stratoulias V, Venero JL, Tremblay M, Joseph B. Microglial subtypes: diversity within the microglial community. The EMBO Journal. 2019 Sep 2;38(17):e101997. 31373067 10.15252/embj.2019101997 PMC6717890

[6] Garcia-Reitboeck P, Phillips A, Piers TM, Villegas-Llerena C, Butler M, Mallach A, Human Induced Pluripotent Stem Cell-Derived Microglia-Like Cells Harboring TREM2 Missense Mutations Show Specific Deficits in Phagocytosis. Cell Reports. 2018 Aug;24(9):2300–11. 30157425 10.1016/j.celrep.2018.07.094 PMC6130048

[7] Hanger B, Couch A, Rajendran L, Srivastava DP, Vernon AC. Emerging Developments in Human Induced Pluripotent Stem Cell-Derived Microglia: Implications for Modelling Psychiatric Disorders With a Neurodevelopmental Origin. Front Psychiatry. 2020 Aug 11;11:789. 32848951 10.3389/fpsyt.2020.00789 PMC7433763

[8] Mrza MA, He J, Wang Y. Integration of iPSC-Derived Microglia into Brain Organoids for Neurological Research. IJMS. 2024 Mar 9;25(6):3148. 38542121 10.3390/ijms25063148 PMC10970489

[9] De Strooper B, Karran E. The Cellular Phase of Alzheimer’s Disease. Cell. 2016 Feb;164(4):603–15. 26871627 10.1016/j.cell.2015.12.056

[10] Long JM, Holtzman DM. Alzheimer Disease: An Update on Pathobiology and Treatment Strategies. Cell. 2019 Oct;179(2):312–39. 31564456 10.1016/j.cell.2019.09.001 PMC6778042

[11] Claes C, Danhash EP, Hasselmann J, Chadarevian JP, Shabestari SK, England WE, Plaque-associated human microglia accumulate lipid droplets in a chimeric model of Alzheimer’s disease. Mol Neurodegeneration. 2021 Dec;16(1):50. 10.1186/s13024-021-00473-0 PMC8305935 34301296

[12] Dräger NM, Sattler SM, Huang CTL, Teter OM, Leng K, Hashemi SH, A CRISPRi/a platform in human iPSC-derived microglia uncovers regulators of disease states. Nat Neurosci. 2022 Sep;25(9):1149–62. 35953545 10.1038/s41593-022-01131-4 PMC9448678

[13] Brase L, You SF, D’Oliveira Albanus R, Del-Aguila JL, Dai Y, Novotny BC, Single-nucleus RNA-sequencing of autosomal dominant Alzheimer disease and risk variant carriers. Nat Commun. 2023 Apr 21;14(1):2314. 37085492 10.1038/s41467-023-37437-5 PMC10121712

[14] Sun N, Victor MB, Park YP, Xiong X, Scannail AN, Leary N, Human microglial state dynamics in Alzheimer’s disease progression. Cell. 2023 Sep;186(20):4386–4403.e29. 37774678 10.1016/j.cell.2023.08.037 PMC10644954

[15] Hao Y, Stuart T, Kowalski MH, Choudhary S, Hoffman P, Hartman A, Dictionary learning for integrative, multimodal and scalable single-cell analysis. Nat Biotechnol. 2024 Feb;42(2):293–304. 37231261 10.1038/s41587-023-01767-y PMC10928517

[16] Hafemeister C, Satija R. Normalization and variance stabilization of single-cell RNA-seq data using regularized negative binomial regression. Genome Biol. 2019 Dec;20(1):296. 31870423 10.1186/s13059-019-1874-1 PMC6927181

[17] Shannon CE. A Mathematical Theory of Communication. Bell System Technical Journal. 1948 Jul;27(3):379–423.

[18] Zimmerman KD, Espeland MA, Langefeld CD. A practical solution to pseudoreplication bias in single-cell studies. Nat Commun. 2021 Feb 2;12(1):738. 33531494 10.1038/s41467-021-21038-1 PMC7854630

[19] He L, Davila-Velderrain J, Sumida TS, Hafler DA, Kellis M, Kulminski AM. NEBULA is a fast negative binomial mixed model for differential or co-expression analysis of large-scale multi-subject single-cell data. Commun Biol. 2021 May 26;4(1):629. 34040149 10.1038/s42003-021-02146-6 PMC8155058

[20] Supek F, Bošnjak M, Škunca N, Šmuc T. REVIGO Summarizes and Visualizes Long Lists of Gene Ontology Terms. Gibas C, editor. PLoS ONE. 2011 Jul 18;6(7):e21800. 21789182 10.1371/journal.pone.0021800 PMC3138752

[21] Chen S, Chang Y, Li L, Acosta D, Li Y, Guo Q, Spatially resolved transcriptomics reveals genes associated with the vulnerability of middle temporal gyrus in Alzheimer’s disease. acta neuropathol commun. 2022 Dec 21;10(1):188. 36544231 10.1186/s40478-022-01494-6 PMC9773466

[22] Chen Y, Colonna M. Microglia in Alzheimer’s disease at single-cell level. Are there common patterns in humans and mice? Journal of Experimental Medicine. 2021 Sep 6;218(9):e20202717. 34292312 10.1084/jem.20202717 PMC8302448

[23] Astillero-Lopez V, Villar-Conde S, Gonzalez-Rodriguez M, Flores-Cuadrado A, Ubeda-Banon I, Saiz-Sanchez D, Proteomic analysis identifies HSP90AA1, PTK2B, and ANXA2 in the human entorhinal cortex in Alzheimer’s disease: Potential role in synaptic homeostasis and Aβ pathology through microglial and astroglial cells. Brain Pathology. 2024 Jan 22;e13235. 38247340 10.1111/bpa.13235 PMC11189773

[24] Krasemann S, Madore C, Cialic R, Baufeld C, Calcagno N, El Fatimy R, The TREM2-APOE Pathway Drives the Transcriptional Phenotype of Dysfunctional Microglia in Neurodegenerative Diseases. Immunity. 2017 Sep;47(3):566–581.e9. 28930663 10.1016/j.immuni.2017.08.008 PMC5719893

[25] Friedman BA, Srinivasan K, Ayalon G, Meilandt WJ, Lin H, Huntley MA, Diverse Brain Myeloid Expression Profiles Reveal Distinct Microglial Activation States and Aspects of Alzheimer’s Disease Not Evident in Mouse Models. Cell Reports. 2018 Jan;22(3):832–47. 29346778 10.1016/j.celrep.2017.12.066

[26] Marsh SE, Walker AJ, Kamath T, Dissing-Olesen L, Hammond TR, De Soysa TY, Dissection of artifactual and confounding glial signatures by single-cell sequencing of mouse and human brain. Nat Neurosci. 2022 Mar;25(3):306–16. 35260865 10.1038/s41593-022-01022-8 PMC11645269

[27] Wang S, Sudan R, Peng V, Zhou Y, Du S, Yuede CM, TREM2 drives microglia response to amyloid-β via SYK-dependent and -independent pathways. Cell. 2022 Oct;185(22):4153–4169.e19. 36306735 10.1016/j.cell.2022.09.033 PMC9625082

[28] Spangenberg E, Severson PL, Hohsfield LA, Crapser J, Zhang J, Burton EA, Sustained microglial depletion with CSF1R inhibitor impairs parenchymal plaque development in an Alzheimer’s disease model. Nat Commun. 2019 Aug 21;10(1):3758. 31434879 10.1038/s41467-019-11674-z PMC6704256

[29] Wes PD, Sayed FA, Bard F, Gan L. Targeting microglia for the treatment of Alzheimer’s Disease. Glia. 2016 Oct;64(10):1710–32. 27100611 10.1002/glia.22988

[30] Sun J, Nan G. The extracellular signal-regulated kinase 1/2 pathway in neurological diseases: A potential therapeutic target (Review). International Journal of Molecular Medicine. 2017 Jun;39(6):1338–46. 28440493 10.3892/ijmm.2017.2962 PMC5428947

[31] Porro C, Pennella A, Panaro MA, Trotta T. Functional Role of Non-Muscle Myosin II in Microglia: An Updated Review. IJMS. 2021 Jun 22;22(13):6687. 34206505 10.3390/ijms22136687 PMC8267657

[32] Gitik M, Reichert F, Rotshenker S. Cytoskeleton plays a dual role of activation and inhibition in myelin and zymosan phagocytosis by microglia. FASEB j. 2010 Jul;24(7):2211–21. 20179145 10.1096/fj.09-146118

[33] Liu C, Xu S, Liu Q, Chai H, Luo Y, Li S. Identification of immune cells infiltrating in hippocampus and key genes associated with Alzheimer’s disease. BMC Med Genomics. 2023 Mar 13;16(1):53. 36915078 10.1186/s12920-023-01458-2 PMC10009990

[34] Zenaro E, Pietronigro E, Bianca VD, Piacentino G, Marongiu L, Budui S, Neutrophils promote Alzheimer’s disease–like pathology and cognitive decline via LFA-1 integrin. Nat Med. 2015 Aug;21(8):880–6. 26214837 10.1038/nm.3913

[35] Fiala M, Liu QN, Sayre J, Pop V, Brahmandam V, Graves MC, Cyclooxygenase-2-positive macrophages infiltrate the Alzheimer’s disease brain and damage the blood–brain barrier. Eur J Clin Investigation. 2002 May;32(5):360–71. 10.1046/j.1365-2362.2002.00994.x 12027877

[36] Hultman K, Strickland S, Norris EH. The APOE ε4/ε4 Genotype Potentiates Vascular Fibrin(Ogen) Deposition in Amyloid-Laden Vessels in the Brains of Alzheimer’s Disease Patients. J Cereb Blood Flow Metab. 2013 Aug;33(8):1251–8. 23652625 10.1038/jcbfm.2013.76 PMC3734776

[37] Illes P, Rubini P, Ulrich H, Zhao Y, Tang Y. Regulation of Microglial Functions by Purinergic Mechanisms in the Healthy and Diseased CNS. Cells. 2020 Apr 29;9(5):1108. 32365642 10.3390/cells9051108 PMC7290360

